# News Items

**Published:** 1886-01

**Authors:** 


### NEWS ITEMS.

INTERNATIONAL CONGRESS.—SPECIAL ANNOUNCEMENT.—The Executive Committee of the Ninth International Medical Congress, to be held in the city of Washington, D. C., commencing on the first Monday in September, 1887, having accepted, under Rule 10 of the Committee on Preliminary Organization, the charge of the business of the Congress, hereby give notice to the members of the medical profession that they have been actively engaged upon, and have now nearly completed, the arrangements for this meeting; and they anticipate the hearty coöperation of the profession everywhere in developing this great scientific and humanitarian assembly.

By order of the Executive Committee.

HENRY H. SMITH, M. D., Philadelphia,
*Chairman of Executive Committee.*

NATHAN S. DAVIS, M. D., LL. D.,
*Secretary-General of Ninth Int. Med. Congress.*

*Chicago, Nov. 24, 1885.*

---

CHICAGO WATER-SUPPLY.—A systematic observation of the varying character of the water-supply of Chicago was begun early in September, under the direction of the Illinois State Board of Health. The chemical examinations by Prof. Long are made weekly, and coupled with the meteorological conditions, recorded and furnished by the U. S. Signal Service for this purpose, will result in a scientific determination of the relation of the purity of the water-supply to the movement of the sewage contents of the Chicago river.

---

CHINESE-AMERICAN DOCTORS.—The *Times*, of Los Angeles, California, contains the advertising cards of several Chinese physicians, as follows :

**Dr. Yep Bow Yuen**

HAS PRACTICED FOR MANY YEARS with the well-known physician, Dr. WON TONG WING, in Canton, and is now opening an office at No. 104 Upper Main st. Can be found at office all hours, day and night.

---

**Dr. Wong Him,**

PHYSICIAN AND SURGEON — THE greatest Chinese Doctor. Makes a specialty of and cures Consumption, Diseases of the Head, Throat, Lungs, Liver, Stomach, Blood, etc. Office No. 28 Upper Main st., Los Angeles. P. O. Box 562.

---

**Dr. Ngut Chow.**

DR. WONG HAVING GONE TO CHINA, Dr. Ngut Chow, a graduate from the same universities at Hong Kong and Canton, will administer to the patients of Dr. Wong, having been tested and found able by him. No. 125 Upper Main st., Los Angeles, Cal.
DR. WONG.

Presumably these men practice only among their own countrymen, and are as ignorant as the other native physicians of China. The universities mentioned in one card are medical colleges of the lowest possible grade. A large part of the treatment taught consists in scarifying or acupuncture of various parts of the body. A life-sized manikin is used in teaching the different portions of the body to be so treated in the principal diseases. So limited is the knowledge of anatomy and surgery that only the most trivial minor operations are attempted.

---

THE following words formed the conclusion of a paper lately read by the Rev. Clinton Locke before one of the Chicago clubs, on the question: "How Does a Doctor Look to a Clergyman?"

"I do really believe that on the face of the earth there is not a class of men more self-sacrificing, more desirous to benefit their fellow-creatures, more regardless of the mere getting of money and attaining of place than the members of the medical profession. I do not believe any of you, unless you have really examined the subject, have any idea of the amount of charitable work done by nearly every doctor in this city. Their time is often extremely valuable. People are often willing to give them any price to come to them, and yet they will cheerfully give hours and hours to the care of the poor, and by the bedsides of families of poor people from whom they know they will receive no remuneration. They are often exposed to very mean treatment. It is well known that they seldom take legal measures to secure their pay, and of this great advantage is taken. Babies do not come into the world with the label 'C. O. D.' pasted on them, nor can a doctor say to a man squirming in a bilious colic, 'Pay me that thou owest, or squirm indefinitely.' Yet, with all this before him, I do not believe any decent doctor ever refused to lend his aid to any suffering creature, no matter whether he was paid, or not paid. I have often known the doctors of St. Luke's Hospital to get up out of their beds in the middle of the coldest nights and hurry to the hospital to relieve, if possible, the agony of some pauper who would not even thank him. All honor to the noble guild of Æsculapius. No men deserve more at the hands of their fellow-citizens than they. Every man here knows what his family doctor is to him, how close a friend, how kind a counsellor, how trusty and how faithful. Think what the world owes of comfort and rescue from pain to the unceasing studies, the high-souled efforts of the doctors of England and America. How little they gain by it. How immeasurably we have entered into the fruit of their labors. Let me sum this up, for my time is out. Doctors are the best friends, the pleasantest company, the most generous, and the most self-sacrificing of any order of men it has been my lot to know."

